# Millifluidic magnetophoresis-based chip for age-specific fractionation: evaluating the impact of age on metabolomics and gene expression in yeast†

**DOI:** 10.1039/d4lc00185k

**Published:** 2024-05-13

**Authors:** L. Wittmann, M. Eigenfeld, K. Büchner, J. Meiler, H. Habisch, T. Madl, R. Kerpes, T. Becker, S. Berensmeier, S. P. Schwaminger

**Affiliations:** a TUM School of Engineering and Design, Chair of Bioseparation Engineering, Technical University of Munich Boltzmannstr. 15 85748 Garching Germany s.schwaminger@tum.de; b TUM School of Life Science, Chair of Brewing and Beverage Technology, Technical University of Munich Weihenstephaner Steig 20 85354 Freising Germany roland.kerpes@tum.de; c Otto-Loewi Research Center, Division of Medicinal Chemistry, Medical University of Graz Neue Stiftingtalstr. 6 8010 Graz Austria; d BioTechMed-Graz Mozartgasse 12/II 8010 Graz Austria sebastian.schwaminger@medunigraz.at; e Munich Institute of Integrated Materials, Energy and Process Engineering, Technical University of Munich Lichtenberstr. 4a 85748 Garching Germany

## Abstract

A novel millifluidic process introduces age-based fractionation of *S. pastorianus* var. *carlsbergensis* yeast culture through magnetophoresis. *Saccharomyces* yeast is a model organism for aging research used in various industries. Traditional age-based cell separation methods were labor-intensive, but techniques like magnetic labeling have eased the process by being non-invasive and scalable. Our approach introduces an age-specific fractionation using a 3D-printed millfluidic chip in a two-step process, ensuring efficient cell deflection in the magnetic field and counteracting magnetic induced convection. Among various channel designs, the pinch-shaped channel proved most effective for age differentiation based on magnetically labeled bud scar numbers. Metabolomic analyses revealed changes in certain amino acids and increased NAD^+^ levels, suggesting metabolic shifts in aging cells. Gene expression studies further underlined these age-related metabolic changes. This innovative platform offers a high-throughput, non-invasive method for age-specific yeast cell fractionation, with potential applications in industries ranging from food and beverages to pharmaceuticals.

## Abstract 2

A novel millifluidic magnetophoresis approach can be used to fractionate yeast cells according to their individual cell age. Old and young cells show different behavior in gene expression and their metabolome.

## Graphical abstract

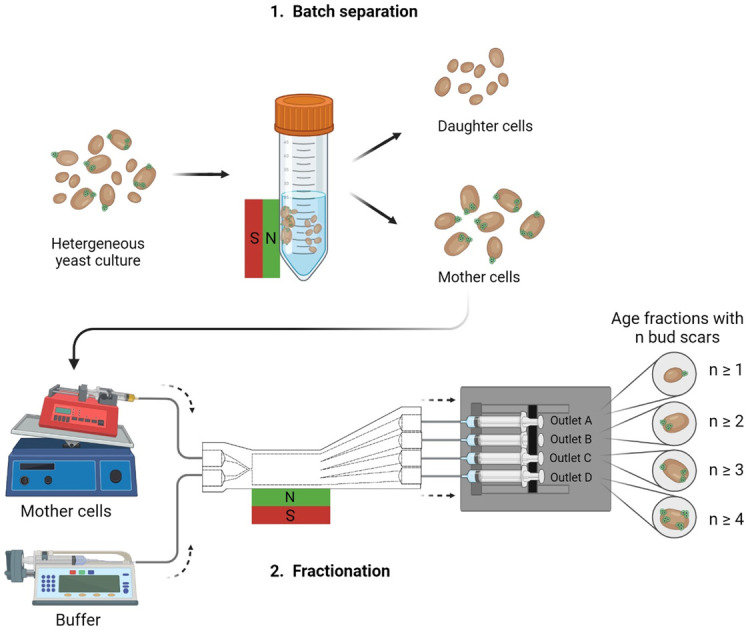

## A Introduction

*Saccharomyces* yeast is a valuable model organism for aging research, offering insights into two distinct aging processes: chronological aging, which is defined by the survival time of the cell, and replicative aging, characterized by the number of division events a cell undergoes before reaching senescence. Senescence, a key aging marker, impedes cellular repair and is linked to age-related diseases.^1^ The process of asymmetric cell division in yeast, wherein mother cells generate a finite number of daughter cells, presents a unique opportunity to gain a deeper understanding of these aging dynamics.^2^

Numerous studies have investigated cellular aging by analyzing heterogeneous cultures or relying on the variable correlation between cell size and age, often employing a sucrose gradient method.^3–8^ However, the direct link between cell age, its metabolome, and gene expression remains a topic of debate, because of the lack of methodology, specifically sorting cells by their replicative age. Recent studies have shown that external factors, such as growth rate and stressors like formic acid, influence yeast metabolic reactions, impacting both oxidative stress response and protein biosynthesis.^6,7^ Correia-Melo *et al.* highlighted the connection between metabolism and chronological aging, marked by shifts in intracellular metabolic processes and signaling pathways.^8^ Thus, there is a need for a reliable, age-specific fractionation method of yeast cells to advance aging research concerning the replicative lifespan.

Historically, age-based cell separation was labor-intensive, relying on microdissection.^2^ Modern microfluidic platforms, leveraging cell size differences or surface adhesion, have simplified this process.^9–11^ However, these techniques can be invasive, potentially compromising age-analysis accuracy, and often lack scalability, making growth and omics studies difficult.^12,13^ High-throughput technologies, like magnetic labeling, facilitate collecting up to 106 cells per mL.^14–16^ The method developed by Hendrickson *et al.* involves biotinylation of the yeast cell wall; however, this modification is not inherited by daughter cells. Consequently, mother cells can bind to streptavidin-coated magnetic beads and are retained by a magnet.^15^ However, a fractionation based on distinct age remains absent.

Employing a laminar flow combined with an external magnetic field offers the advantages of being non-invasive and efficient compared to other methods.^17^ The primary forces in this process are hydrodynamic drag and magnetophoretic force acting on the entities labeled with magnetic nanoparticles (MNP),^18,19^ with the latter defined as,1
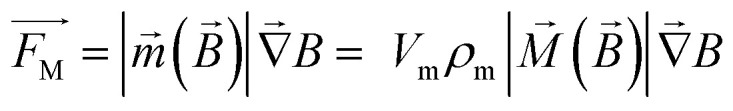
Where 
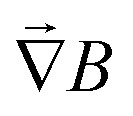
 is the magnetic field gradient within the magnetic field strength *B*, inducing a magnetic dipole moment 
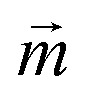
. It depends on the volume (*V*_m_) and density (*ρ*_m_) of the magnetic entity, as well as the volumetric magnetization in solution 
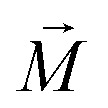
. Beyond this classical model, two phenomena enhance magnetophoretic velocity, substantially impacting a magneto-responsive fractionation. The first, cooperative magnetophoresis, arises from the combined motion of interacting magnetic dipoles (Eqn S1 and S2†).^18,20,21^ The second is a fluid dynamics instability caused by the magnetic field gradient (Eqn S3†).^19,22,23^ The hydrodynamic drag force originates from the Stokes equations, with particles in laminar flow aligning with fluid streamlines based on size due to the inertial force.^24^

**Fig. 1:**
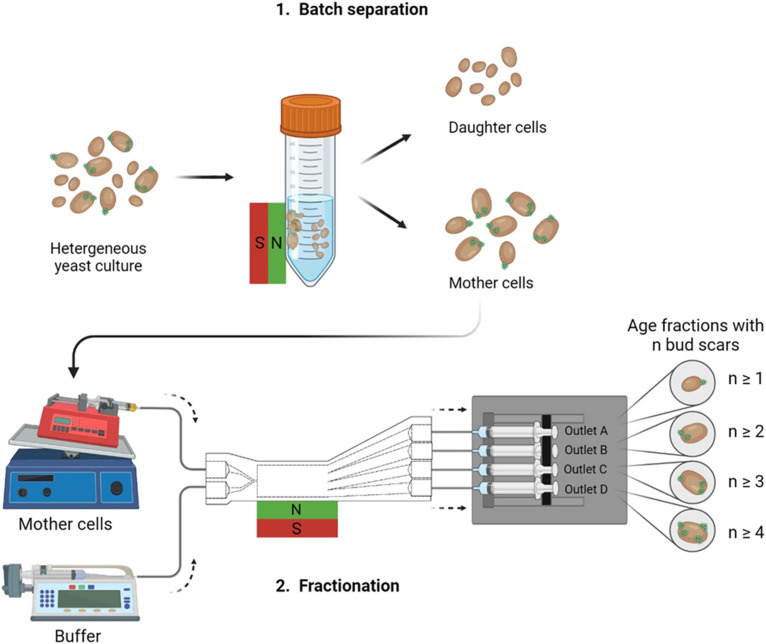
Millifluidic yeast cell fractionation scheme. The age-dependent fractionation process consisted of a batch separation removing the young, unlabeled daughter cells. The magnetically labeled mother cells were introduced into the chip for further age-based fractionation. Three chip geometries were tested (compare Fig. 4a). Chip outlet A was the furthest away from the magnet; chip outlet D was the nearest one to magnet, consisting of the oldest cells with the highest bud scar number. Further details about the set-up are given in section B.

**Fig. 2:**
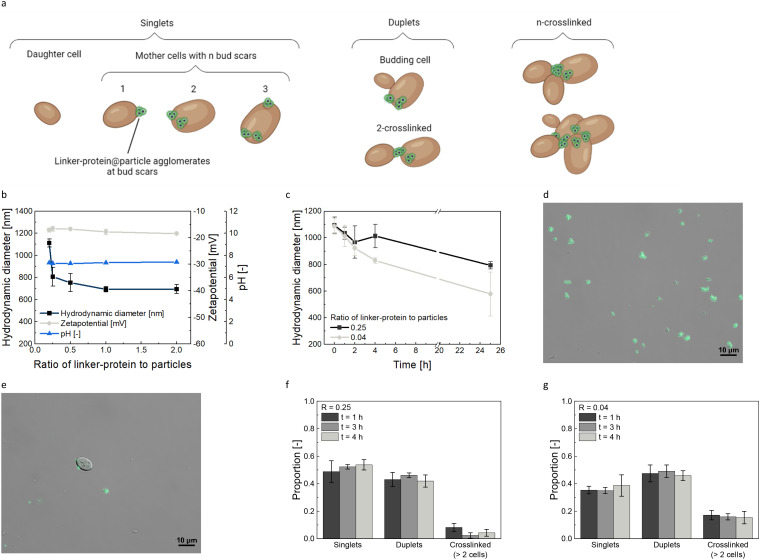
Yeast cell-nanoparticle binding analysis. Schematic illustration of the magnetic labeling of yeast cell bud scars. Cells possessing n bud scars could bind n agglomerates. Unlike single cells, duplets are composed of either budding cells or a pair of cells crosslinked through the agglomerates. ‘n-crosslinked cells’ denotes an assembly of more than two cells (a). Hydrodynamic diameter, zeta potential, and pH measurements for different linker-protein to particle ratios in a pH 7.3 buffer after 24 h. While the linker-protein concentration was constant at *c*_Linker-protein_ = 0.02 g L^−1^, the particle concentration varied between *c*_Particle_ = 0.1 and 0.01 g L^−1^ (b). Hydrodynamic diameter over time for particles combined with linker-protein post-incubation in buffer was evaluated for two linker-protein-particle ratios. The particle concentrations were *c*_Particle_ = 0.5 and 0.08 g L^−1^ with a consistent linker-protein concentration of *c*_Linker-protein_ = 0.02 g L^−1^ (c). Light microscopy image of agglomerates formed by a ratio of linker-protein to particles of *R* = 0.25 (d). Light microscopy visualization displays the specific binding of the particles labeled yeast cell bud scars (3.17 × 10^6^ cells per mL) *via* the linker-protein at a *R* = 0.25 ratio (e). Crosslinkage analysis of magnetically labeled yeast cells (3.17 × 10^6^ cells per mL). The relative distribution of singlets (single cells), duplets (two cells), and crosslinked cells (agglomerates of more than two cells) is presented, corresponding to the ratios as in (b) and observed at 1, 3, and 4 hours after incubation (based on *n* = 450 cells) (f) and (g). All data plots include the standard deviation from a triplicate measurement.

Integrating size separation with magnetic bud scar labeling^25,26^ offers a promising method for age-based yeast cell fractionation (Fig. 1). Yeast cell bud scars are magnetically labeled using a linker-protein, giving each cell an age-dependent susceptibility (Fig. 2a).^25,26^ In designing this millifluidic magnetophoretic process, several critical factors must be considered:

1. The particle concentration has to be regulated to avoid crosslinkage and undesired magnetically induced convective motion of unlabeled cells while ensuring all bud scars are covered.

2. The agglomerates' susceptibility, meaning size, must be substantial enough for magnetic manipulation within the chip. From eqn (1), the magnetic response is contingent upon the magnetic dipole moment of the magnetic entities, influenced by the agglomerates' size and concentration.^18^

3. The agglomerate size must be consistent throughout the process.

Our study introduces a 3D-printed chip that separates yeast cells by bud scar count, advancing the microfluidic single-cell method to a high-throughput millifluidic platform, giving insights into metabolomics and gene expression.

## B Methods

### A Synthesis of ethylenediaminetetraacetic acid-functionalized silica coated iron oxide nanoparticles

Iron oxide nanoparticles were synthesized *via* co-precipitation^27,28^ and subsequently coated with silica to increase the colloidal stability. As previously described, the MNPs were further functionalized with EDTA by an amide bonding.^25^ Briefly, 179 mg of iron oxide nanoparticles were suspended in 100 mL of Millipore water, ultrasonicated on ice (3 min, 20%, 10 sec on, 15 sec off, 20 kHz, Branson Ultrasonics), mixed with citric acid (Carl Roth, *c* = 0.029 mol L^−1^, *V* = 100 mL, ultrasonicated), and ultrasonicated as before. After 15 min incubation, the pH was adjusted to 11 using tetramethylammonium hydroxide (Sigma Aldrich). The mixture was transferred into a nitrogen-evacuated flask, combined with ethanol (*V* = 2.72 L, 99%, VWR), Millipore water (*V* = 0.72 L), ammonia (*V* = 0.18 L, 25%, Carl Roth), and the prepared MNP solution (*V* = 0.18 L), with the ethanol being critical to dispense the MNPs and minimize agglomerate size. The synthesis was started by adding 6.94 mL of tetraethyl orthosilicate (TEOS, Sigma Aldrich), forming the silica shell around the MNPs. The reaction was conducted at 4 °C and was continuously dispersed *via* ultrasonication (45 kHz, VWR). 1.984 mL (3-Aminopropyl)triethoxysilane (APTES, VWR) was added an hour later to introduce amine groups for the later EDTA functionalization. After another hour, particles were washed by centrifugation (1×, 4600 × *g*, 30 min) and magnetically (min. 7×) with ethanol until pH = 9.5–9.7 was reached. The washing was continued with degassed Millipore water (minimum 3×) to reach conductivity <150 μS cm^−1^. Ultrasonication (5 min, 20%, 10 sec on, 15 sec off, Branson Ultrasonics) dispersed the MNPs before under nitrogen storage at 4 °C. The concentration was analyzed *via* gravimetry by drying 300 μL of MNP suspension overnight. The MNPs were functionalized with EDTA *via* amide bonding in a subsequent synthesis. Therefore, 100 mg particles were mixed with EDTA (*c* = 0.075 mol L^−1^, *V* = 100 mL, Carl Roth) and ultrasonicated (132 kHz, Sonorex) at 60 °C for 2 hours. Subsequently, the EDTA-functionalized MNPs were washed with degassed Millipore water below a conductivity of 150 μS cm^−1^ and stored under nitrogen at 4 °C. ‘MNP’ refers to the EDTA-functionalized silica shell iron oxide nanoparticles in the following.

The magnetization was analyzed with a superconducting quantum interference device (SQUID) (Quantum Design MPMS XL-7) at 300 K (−50–50 kOe) using a minimum of 10 mg freeze-dried MNPs. The data was fitted by the LangevinMod fit in Origin2020. XRD (STOE Sadi-P) of freeze-dried MNPs was performed using a molybdenum source (0.7093 Å, 1 = 0). For transmission electron microscopy (TEM) measurement (JEOL 1400 plus), 10 μL of 0.1 g L^−1^ MNP solution was dried on a discharged carbon-coated copper grid. The images were analyzed *via* the Software ImageJ, and at least 100 particles were analyzed to obtain the primary particle diameter. Thermogravimetric analysis (TGA) (STA 449C Jupiter) was performed between *T* = 25–700 °C, holding 700 °C for 10 min, using freeze-dried MNPs in a 50 μL aluminum oxide jar. The MNPs were further characterized by Fourier-transform infrared spectroscopy (FT-IR) (Alpha II, Bruker) with a platinum attenuated total reflection module using 24 scans per sample between the wave number range 4000–400 cm^−1^, subtracting the background by the concave rubber band method. The spectra were normalized to the vibration of the magnetite peak at ∼570 cm^−1^. Dynamic light scattering (DLS) and zeta potential measurements (Zetasizer Ultra, Malvern Panalytical) were conducted at a concentration of 1 g L^−1^ (if not stated otherwise) in water or the corresponding buffer at 25 °C. To determine the isoelectric point, the pH was adjusted by HCl or NaOH 24 h and 1 h before the measurement so that the particles could equilibrate. The data was fitted by using the Boltzmann fit in Origin2020. The magnetophoretic sedimentation velocity was measured by the LUMiReader (4532–123, LUM GmbH) at 630 nm (angle = 0°, light factor 1.00, *T* = 25 °C, 300 profiles: interval = 10 s, then 100 profiles: interval = 20 sec). Nickel was analyzed *via* induced coupled plasma optical emission spectroscopy (ICP-OES) (Agilent Technologies 700 series ICP-OES). For the adsorption isotherm to nickel, 1 g L^−1^ of MNPs (ultrasonicated, 3 min, 20%, 10 sec on, 15 sec off) were incubated with nickel chloride hexahydrate (Sigma Aldrich, *c* = 0.05, 0.03, 0.01, 0.005, 0.001, 0.0005 and 0 mol L^−1^) for 2 h at 1000 rpm at *T* = 22 °C. The samples were washed by centrifugation for 10 min at 12 000 × *g* twice with Millipore water and dispersed *via* ultrasonication (15 min) and pipetting. For investigating the kinetic and leaching behavior, samples were taken after 5, 15, 30, 60, and 120 min and 0, 1, 2, and 5 days, respectively, following the washing procedure as described. The samples were prepared for ICP-OES by dissolving 0.48 mL of labeled MNPs in 20% nitric acid for 2 h at 40 °C. After incubation overnight, the final volume was set to 7 mL, always using Millipore water. The calibration curve was generated using a multi-element standard solution (Sigma Aldrich).

### B Yeast cell labeling

The yeast cells were magnetically loaded, as described in Eigenfeld and Wittmann *et al.*^25^ Therefore, yeast cells (*Saccharomyces pastorianus* var. *carlsbergensis* TUM34/70) were grown in 15 mL yeast extract peptone dextrose medium, consisting of 10 g L^−1^ yeast extract (VWR), 20 g L^−1^ peptone (Carl Roth), 20 g L^−1^d-glucose (Merck) at 120 rpm at 22 °C overnight until end-log phase. For labeling, the cells were rebuffered in 20 mM MOPS (pH = 7.3), and 3.17 × 10^7^ cells per mL were incubated with 0.4 g L^−1^ linker-protein (*His6-Sumo-sfGFP-ChBD*),^26^ enabling the specific labeling of the chitin-enriched yeast cell bud scars. After 30 min at 22 °C at 1000 rpm, the samples were washed twice with buffer at 1000 × *g* for 2 min. In the meantime, the MNPs were loaded with 0.01 mol L^−1^ nickel chloride hexahydrate for 15 min to form a chelat complex with EDTA. This allows the nanoparticles to form a coordinative bond to the His-tag of the linker-protein. The washing procedure was performed in buffer, and during the last washing step, the MNPs were concentrated to 2 g L^−1^. Then, the linker-protein labeled yeast cells (*c* = 1.58 × 10^7^ cells per mL) were incubated with the Nickel-loaded MNPs, which have been dispersed by ultrasonication (45 kHz) for 5 min shortly before the mixing (*c* = 0.4 g L^−1^) for 1.5 h at 1000 rpm at 22 °C. That ratio was always kept constant, except stated otherwise (see Fig. 2b and c). The specificity of the nanoparticle binding has been verified before.^25^ Microscopic images are taken by a Zeiss Axio Observer 7, 100× objective using bright field and fluorescent channels. The extinction wavelength was 480 nm and the emission wavelength was 505 nm. The exposure time for the fluorescent image was set to 1000 ms, for the bright field it was automatic.

## C Device design and fabrication

The chip geometries were designed in Autodesk Inventor Professional 2023 and exported as a high-resolution STL file. Subsequently, support structures were added to the printing parts *via* the software Preform Version 3.27.1. The support structure density was 1.0, and the contact point size was set to 0.4 mm, manually removing structures inside the in- and outlets. The parts were printed with a layer of 0.025 mm with clear resin (Formlabs, RS-F2-GPCL-04) using a Form 3B+ stereolithographic (SLA) 3D printer (Formlabs). The flow channels were placed horizontally and twisted in every direction, and the tubing adapters were printed separately and glued into the chips afterward to facilitate the washing process. The printed parts were rinsed manually with 100%-isopropanol (VWR) using syringes or cannulas to remove the residual uncured resin inside the channels. Afterward, they were placed into an isopropanol bath for 2 hours before curing the structures for 30 min at 60 °C in UV light (Form Cure, Formlabs). The channel height was kept constant at 750 μm for all channels (Fig. S5†). First, we designed the rectangular channel, which was on a centimeter scale in length; however, too many convective and diffusive effects occurred. That is why the trapezoidal and pinch-shaped channels were designed with smaller lengths. Existing literature with similar separation processes was used to design the channels.^29–31^ Then, solely yeast cells and only MNPs separately were fractionated to get an indication of the necessary magnetic field strength.

### D Process development

The set-up consisted of a sample syringe pump (Legato 110, kdScientific), a buffer syringe pump (Alaris plus GH, Juaramed), and an outlet pump (A-51133, Havard Apparatus) with a manually constructed multi-syringe adapter. The syringes were connected to the chip *via* printed and commercial (Luer female, Reichelt Chemietechnik) adapters and silicone tubings (*d*_inside_ = 1.3 mm, VWR) to the chip. The chip was first flushed with buffer to start a fractionation process, avoiding bubble formation. Then, the sample pump was started, the pipe was connected to the chip, and the magnet was placed at the defined position. After the equilibration time, the outlet pipes were connected to the running outlet pump. Fraction A refers to the outlet being furthest away from the magnet; fraction D was the one nearest to the magnet. Before each run, the flow channel was flushed and cleaned with buffer to remove bubbles and residual MNPs. For storage, the flow channels were washed with ethanol and water; lastly, the chips were dried with compressed air.

The sedimentation behavior was analyzed by introducing 1.58 × 10^7^ cells per mL of yeast suspension into a syringe, and yeast cell samples were collected *via* the connected sample pipe after *t* = 0, 5, 10, 15, and 20 min for 70 sec at *V̇* = 100 μL min^−1^. The different dispersion methods included no dispersion, a shaker (Eppendorf) at 300 rpm, and a rocker (witeg) at 30 rpm with and without inserting a 5 mm silver sphere into the sample syringe. The yeast cell concentration was analyzed *via* UV-vis at 600 nm (Tecan Infinite M200). Analyzing the sedimentation behavior dependent on the yeast cells' (agglomerate) diameter and time further, the LUMiReader was used at 410 nm (angle = 0°, light factor 1.00, *T* = 22 °C, 350 profiles: interval = 20 s). For yeast, a refraction index (RI) of RI = 1.4 with a density of *δ* = 1.03 kg m^−3^, and for water, a refraction index of RI = 1.327 with a density of *δ* = 997.8 kg m^−3^ and a viscosity of *μ* = 0.95 mPa s was used. To verify that the specificity of the binding and the viability were maintained during dispersion, a magnetically labeled yeast cell culture with and without dispersion *via* the rocker, including the silver sphere, were compared regarding their viability and binding specificity, as described in Eigenfeld and Wittmann *et al.*^25^ The magnetically induced convective motion was investigated using the same particles and yeast cell concentrations under fractionation conditions and a 9 × 9 × 3 cm neodymium-iron-boron magnet. A single experiment refers to the fractionation of only yeast cells or MNPs; for the mixture experiments, yeast cells and MNPs were mixed but without the linker-protein, investigating the independent hydrodynamic motion of each component. The yeast cell number was investigated microscopically by haemacytometry using a Neubauer counting chamber (Marienfeld), and the particle concentration was derived by UV-vis absorbance measurement at 400 nm. The yeast cell absorbance was subtracted *via* a linear equation system, necessitating a measurement and calibration curve for both components at 400 nm and 800 nm. The cell size was determined using microscopic images (Zeiss Axio Observer 7, 20× objective) and the particle analysis function in ImageJ. Single cells were selected by adjusting the circularity to 0.8–1.00.

### E Age-dependent fractionation

The magnetically labeled yeast cells were magnetically separated for 15 min using a 7 × 7 × 3 cm neodymium-iron-boron magnet for the prior batch separation. The daughter cell containing supernatant was removed carefully by pipetting with an adequate small pipette for the small volumes (for 1 mL suspension 30 μL residual volume was left). The magnetically separated mother cells were resuspended in the equivalent buffer volume and incubated for 15 min at 1000 rpm. The sample was drawn into a syringe with the 5 mm silver sphere and placed on the rocker at 30 rpm. The process started as described above with the conditions presented in Table 1. After the experiment, the samples were concentrated (3000 rpm, 10 min), and imidazole was used for MNP elution (*c*_Imidazole_ = 0.26 mol L^−1^ for 3.17 × 10^6^ cells per mL). The samples were vortexed for 1 min and incubated for 10 min at 1000 rpm. Another batch separation followed, removing the eluted MNPs. After 15 min, the supernatant, containing the fractionated cells, was removed and washed twice with buffer (1 min, 17 000 × *g*). If used for cytometric analysis, the cells were loaded again with linker-protein, following the protocol described above. The remaining magnetically separated sample was resuspended in the buffer for subsequent UV-vis analysis at 400 nm to determine the particle concentration. The yeast cell number was determined microscopically (Zeiss Axio Observer 7) by haemacytometry using a Neubauer counting chamber. As described in ref. 25 and 26, a cytometric approach was used to evaluate the bud scar number, applying Gauss fits (detailed information in ESI,† Fig. S7).

**Table 1:** Optimum fractionation conditions to the corresponding chip geometries are presented. The magnet was placed upright with the corresponding distance at the edge of the sample inlet. The exact channel dimensions are detailed in the ESI† (Fig. S5)

Channel geometry	Magnet dimensions [cm]	Magnet distance [cm]	*V̇*_Sample_ [μL min^−1^]	*V̇*_Buffer_ [μL min^−1^]	Equilibration time [s]
Rectangular	9 × 9 × 3	1.4	220	920	23
Trapeze	5 × 1.5 × 1.5	0.5	220	920	0
Pinch-shaped	9 × 9 × 3	0.0	220	1100	0

### F Nuclear magnetic resonance spectroscopy for metabolomics

Yeast cell pellets (cell numbers between 10^6^ and 10^8^ cells per mL) were frozen in liquid nitrogen directly after fractionation and kept at −80 °C until further processing, as previously described in more detail.^32,33^ Pellets were mixed with 600 μL methanol/water (2 : 1) for protein precipitation and stopping of enzymatic reactions. Following homogenization in Precellys tubes filled with 1.4 mm diameter zirconium oxide beads on a Precellys24 tissue homogenizer with 2 cycles of 20 s at 25 °C (Bertin Technologies), transferred in empty 1.5 ml tubes, storage at −20 °C for 1 h, samples were centrifuged at 10 000 rpm for 30 min at 4 °C. Supernatants were transferred into new tubes, lyophilized for 10 h on a Savant SpeedVac SPD210 vacuum concentrator with cooling trap (Thermo Scientific) and finally resuspended in nuclear magnetic resonance (NMR) sample buffer containing 0.08 M Na_2_HPO_4_, 5 mM 3-trimethylsilyl propionic acid-2,2,3,3,-d4 sodium salt (TSP) and 0.04 (w/v)% NaN_3_ in D_2_O, adjusted to 7.4 pH with 8 M HCl and 5 M NaOH.

NMR experiments were performed at 310 K (600 MHz Bruker Avance Neo NMR spectrometer equipped with a TXI 600S3 probe head), applying the one-dimensional Carr–Purcell–Meiboom–Gill (CPMG) pulse sequence (cpmgpr1d, 512 scans, 73 728 points in F1, 12 019.230-Hz spectral width, recycle delay 4 s), with water suppression using presaturation. The ^1^H 1D NMR experiments were recorded and automatically processed (exponential line broadening of 0.3 Hz, phased, and referenced to TSP at 0.0 ppm) by Bruker Topspin software version 4.1.3 (Bruker GmbH). To quantify metabolites of interest by targeted analysis, spectra were imported into Matlab 2014b (Mathworks), aligned, and normalized (by probabilistic quotient normalization^34^) using a state-of-the-art script developed by the group of Prof. Jeremy Nicholson at the Imperial College, London, UK. Processed raw spectra were further processed using an R script for integrating experimentally known chemical shift ranges of metabolites in yeast, cross-checked with commercially available standards, the human and yeast metabolome database, and Chenomx NMR Evaluation Suite 8.2 (Chenomx Inc.). Integrals (in arbitrary units, A.U., proportional to metabolite concentration) were statistically analyzed using MetaboAnalyst 5.0.^35^ Besides univariate ANOVA, multivariate sparse partial least squares discriminant analysis (sPLS-DA) was used to identify metabolites that highly contribute to differences between sample groups. The integrals were normed according to yeast cell concentration.

### G Quantitative polymerase chain reaction (qPCR) for gene expression

For gene expression analysis, we examined yeast cell fractions, including the initial yeast population and fractions from outlets A to D.

To ensure accurate analysis, the samples were centrifugated and treated with trizol reagent for metabolic quenching, effectively stopping metabolic activity and RNA degradation. The treated cells were stored at −80 °C until ribonucleic acid (RNA) isolation was carried out using the Roboclon Universal RNA Purification Kit. We used spectrophotometry to assess RNA concentrations and purity (NanoDrop, Thermo Scientific).

For complementary deoxyribonucleic acid (cDNA) synthesis, we utilized the Maxima H Reverse transcriptase and Ribolock RNAse inhibitor (ThermoFisher), known for its reliability and efficiency. Per preparation, 4 μL RT buffer, 0.5 μL Ribolock, 0.5 μL reverse transcriptase, 0.5 μL RNAse free water, 1 μL Primer oligo DT, and 1 μL DNTPs were used. The primers needed for the real-time reverse transcriptome quantitative polymerase chain reaction (RT-qPCR) were designed using the Clone Manager 9 software from Sci Ed Software. These primers were then synthesized by TIB Molbiol Syntheselabor GmbH (Berlin), and their sequences are available in Tables S2 and S3.† As housekeeping genes, KRE11, UBC6, and TAF10 were used for their stability and consistent expression, making them suitable as references for normalizing the target gene expression levels, as reported by Beugholt *et al.*^36^

During qPCR measurements, a 10 μL final volume was used containing 0.7 μL of DNA template, 0.4 μL of each respective primer, 5 μL of SybrGreen (Biozym), and 3.5 μL of RNase-free water.

To analyze gene expression, we performed real-time RT-qPCR and evaluated the expression stabilities using the geNorm algorithm based on *M* and *V* values. For this analysis, we employed the qBase+ software (Biogazelle), as proposed by Vandesompele *et al.* in 2002.^37^ The C_q_ values from RT-qPCR were then imported into qBase+ software for further examination and comprehensive evaluation of gene expression patterns. The Minimum information for publication of quantitative real-time polymerase chain reaction experiments (MIQE) guidelines are detailed in Table S4.†

## C Results

### A Process-relevant characterization of binding and agglomeration behavior of the yeast@linker-protein@particle complex

Achieving continuous high separation selectivities necessitates the understanding of concentration and time dependence of the linker-protein@particle agglomerate formation (particle characterization in the ESI† (Fig. S1), the term MNP/particle refers to the final ethylenediaminetetraacetic acid (EDTA)-functionalized silica shell iron oxide nanoparticles). Fig. 2b illustrates the hydrodynamic diameter, zeta potential, and pH values for varying particle concentrations, maintaining a consistent linker-protein concentration during labeling.^25,38^ As the ratio R of linker-protein to particle increases from 0.2 to 2, the hydrodynamic diameter of the linker-protein@particle agglomerates diminishes from 1112.0 ± 36.2 nm to 695.6 ± 40.0 nm. Schwaminger *et al.* discussed the formation of large clusters composed of small-sized protein-particle agglomerates.^38^ Hence, adding protein acts as ‘glue’, instigating agglomeration through hydrophobic interactions. However, upon reaching a specific linker-protein to MNP ratio, the MNP surface becomes saturated, and the bridging interaction is repressed by repulsive forces,^39,40^ evidenced by the zetapotential decrease from −17.1 ± 0.8 mV (*R* = 0.2) to −18.4 ± 0.2 mV (*R* = 2). In addition to concentration, time significantly influences the agglomeration dynamics. For consistent magnetophoretic deflection in the chip, sustaining a stable hydrodynamic diameter of the linker-protein@particle agglomerate is crucial. At a ratio of *R* = 0.04, the hydrodynamic diameter steadily decreases from 1086.7 ± 58.6 nm post-incubation to 577.5 ± 165.1 nm over 25 h, as illustrated in Fig. 2c. Conversely, for a higher ratio of *R* = 0.25, uniform agglomerates of 793.3 ± 26.4 nm are formed after the same duration. While still debatable, protein corona formation is generally agreed to involve the rapid formation of an initial protein monolayer, with subsequent changes in particle agglomeration over time. Still, a higher ratio promotes stabilization.^40–43^ Finding a balance between fractionation process duration and agglomerate uniformity, a ratio of *R* = 0.25 emerges as best because the agglomerates' size remains relatively constant, around 1000 nm for the initial five hours (microscopic visualization in Fig. 2d). The age-dependent magnetic yeast labeling is depicted in Fig. 2e, and Eigenfeld and Wittmann *et al.* previously confirmed its specificity through microscopic and cytometric analysis using the same ratio of *R* = 0.25.^25^ As can be seen in Fig. 2d, the MNPs are covered completely with the linker-protein avoiding repulsive forces between the yeast cells and the MNPs. Nevertheless, the maintenance of the binding could not be verified with the existing analytical methods, which could negatively influence the subsequent selectivity of the fractionation. The dependence of crosslinkage among magnetically labeled yeast cells on used MNP concentration is emphasized in Fig. 2f and g. At a ratio *R* = 0.04, the single cells (singlets) account for approximately 0.35 of the total. In contrast, crosslinked cells (cell agglomerates of more than two cells) have a proportion of about 0.18, being time-independent. In contrast, for an increased ratio of *R* = 0.25, singlets comprise around a proportion of 0.5, and crosslinked cells are reduced to less than 0.10, again showing time-independence. For both concentrations, duplets, primarily budding cells (Fig. S2a†), represent 0.45–0.50 of the total since cells from the terminal exponential growth phase were used.

The subsequent process employs a consistent ratio of linker-protein to particle of *R* = 0.25, which ensures optimal MNP agglomerate size and reduced crosslinkage. Additionally, the agglomerates are microscopically visible, correspond to the bud scar size,^25,44^ and the labeled yeast cells can be magnetically manipulated easily.

### C Development of a magneto-responsive fractionation process

Upon entering the chip, the magnetically labeled cells are subjected to an inhomogeneous magnetic field. The resulting magnetophoretic motion is accelerated due to interparticle- and hydrodynamic interactions with the surrounding fluid, known as cooperative and convective magnetophoresis.^18,20–23^ The propensity of these interparticle interactions is quantified by the aggregation parameter (Eqn S1 and S2†) with a determined value of N* = 0.01, further specified in the ESI,† for N* > 1, particles would agglomerate, leading to crosslinkage. However, the MNPs used in our study, encapsulated in a silica shell with a low saturation magnetization of 13.1 emu g^−1^, are less prone to magnetic dipole coupling. This encapsulation enhances their performance in the application, although the induced convective motion by hydrodynamic interaction remains inevitable. It is characterized by the magnetic Grash of number **Gr**_**m**_ (Eqn S3†), calculated as **Gr**_**m**_ = 603.2, indicating a strong regime of induced convective motion (**Gr**_**m**_ ≫ 1).

**Fig. 3:**
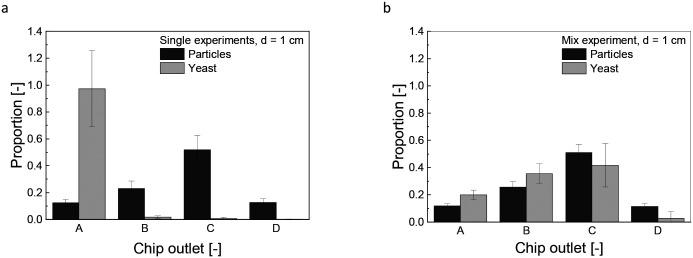
Single fractionation experiments for solely yeast and particles in the rectangular geometry (a). Fractionation experiment for yeast-particle mixture without linker-protein in the rectangular channel (b). Chip outlet A was the furthest away from the magnet; chip outlet D was the nearest one to magnet, and the magnet distance was *d* = 1 cm. The relation between the magnetic field strength and magnet distance is given in Fig. S3 d. *V̇*_Sample_ = 220 μL min^−1^, *V̇*_Buffer_ = 920 μL min^−1^, *c*_Particle_ = 0.4 g L^−1^, cell number = 1.58 × 10^7^ cells per mL. All data plots include the standard deviation from a triplicate measurement.

The fractionated heterogeneous yeast culture contains magnetically labeled mother cells and non-magnetically labeled daughter cells. Preliminary studies were conducted to understand the influence of this convective motion on separation efficiency. These experiments, which involved solely yeast cells or MNPs (Fig. 3a), were compared with trials using a mixture of both but without linker-protein (Fig. 3b). So, the direct influence of the MNPs on the non-magnetic unlabeled yeast or daughter cells is analyzed. Fig. 3a reveals that yeast cells are unaffected by the magnetic field without MNPs. In contrast, MNPs experience a magnetophoretic force, diverting them significantly to chip outlet C with a mean proportion of 0.51 ± 0.06. The main particle fraction does not accumulate in chip outlet D because an increased magnetic field gradient would cause the particles to adhere to the chip wall, preventing flow into the outlet channels. In mixed solution, the deflection pattern of the MNPs remains constant as in solely conditions, but the non-magnetic yeast cells are carried along the MNP motion (Fig. 3b). This magnetic-induced convective motion would compromise the separation efficiency of the fractionation process with a heterogeneous, labeled culture, suggesting that daughter cells might co-migrate with mother cells. Additionally, it can be assumed that not all nanoparticle agglomerates bind to the yeast cells, which increases the effect of magnetically induced convection. Increasing the hydrodynamic force by an increased buffer flow was not purposeful, as the magnetophoretic force reached a threshold beyond which it could not surmount the hydrodynamic drag force (Fig. S3b and e†). Elevating the magnetic field further intensifies the magnetically induced hydrodynamic force (Fig. S3c and f, Eqn S2†). Microfluidic techniques using magnetic labeling are widely used for separation processes. Robert *et al.* separated magnetic macrophages from non-magnetic monocytes, having an iron content of 0.02 g L^−1^, a concentration consistent with our study. They minimized monocyte drag by increasing buffer velocity and counteracting the magnetophoretic force.^45^ Notably, their study employed a lower magnetic field strength of 0.26 T, in contrast to our 0.41 T (compare Fig. S3f†). The characteristic length of the system profoundly affects the induced convective motion (Eqn S3†). This is crucial because they used a microfluidic channel, while this study used centimeter-scale millifluidic channels. However, a high-throughput fractionation necessitates a millifluidic flow channel geometry, high concentrations, flow rates, and a stronger applied magnetic field. Yet, the convective motion of non-magnetic entities remains an inherent challenge in these settings. Although working with a microfluidic chip, Lin *et al.* presented a process-oriented solution, proposing a two-stage method for separating magnetically labeled white from red blood cells, effectively separating the co-migrating red blood cells.^46^

Consequently, this study adopts a two-step process for the age-based fractionation. A batch separation was performed outside the chip, separating non-magnetic daughter cells from magnetically labeled mother cells.^15,25^ Subsequently, the mother cells were subjected to age-based fractionation within the chip (Fig. 1).

### D Channel design for the age-based fractionation of a yeast cell culture

**Fig. 4:**
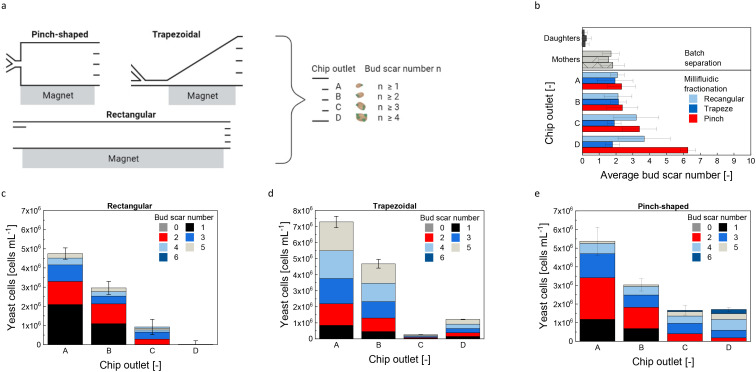
Scheme of different chip designs for age-dependent fractionation (a). Average bud scar number for different fractions with different flow channels (b). Yeast cell concentration for the different fractions and corresponding bud scar numbers for the rectangular (c), the trapezoidal (d), and pinch-shaped channel (e) with *c*_Particle_ = 0.4 g L^−1^, cell number = 1.58 × 10^7^ cells per mL. Error bars represent the standard deviation of the triplicate measurement. Outlet A was the furthest away from the magnet; outlet D was the nearest to the magnet. Daughter and mother cells refer to the prior batch separation.

Three different millifluidic flow channels were evaluated to differentiate age based on bud scar numbers, as presented in Fig. 4a. The first channel employed a traditional rectangular design, commonly utilized in numerous microfluidic applications.^31,47–50^

The second channel featured a trapezoidal design, enhancing the exposure to the magnetic field gradient due to its widened shape.^30,51^ A pinch-shaped geometry, the third channel, combined magnetic with intertial sorting,^19,29,52,53^ providing an additional separation criterion since yeast cells increase in size during their replicative lifespan.^4,25^ As can be seen in the average bud scar number in Fig. 4b, the batch-separated fraction (pre-fractionation, see Fig. 1 step 1) only consists of young cells having a bud scar number of almost zero, a result consistent across all channel geometries.

After the batch separation, the mother cells exhibit an average bud scar number of approximately 1.7 across all three chip geometries. For the subsequent fractionation in the rectangular chip (Fig. 4c), the average bud scar count increases from 2.08 ± 0.41 at chip outlet A (furthest from the magnet) to 3.67 ± 1.55 at chip outlet D (closest to the magnet). This high variation in the oldest fraction is attributed to the low cell concentration of 8.73 × 10^3^ ± 8.00 × 10^3^ cells per mL at that outlet, making age analysis difficult. The chip's extended length in the millifluidic design results in prolonged residence time for labeled cells, promoting increased convective mixing motion and diffusion.^18,23,54^ These effects reduce the separation selectivities shown in Table S1.† Only in fraction C cells with two or more bud scars are enriched. Most cells reach outlet A, minimally influenced by the magnetophoretic force due to buffer flow dilution, which reduces cell concentration and magnetic responsiveness (eqn (1)). In the trapezoidal channel, the average bud scar count remains consistent at approximately two across all outlets (Fig. 4d). Analysis of cell fractions in Fig. 4e indicates a lack of age fractionation, with bud scars ranging from one to five at every outlet. Notably, almost no cells are collected in fraction C. For the pinch-shaped channel, the average bud scar number increases from 2.33 ± 0.82 in fraction A to 6.27 ± 0.45 in fraction D, suggesting enrichment of old cells. Fig. 4e confirms this, as fractions C and D contain cells with two or more bud scars at concentrations of 1.66 × 10^6^ ± 4.81 × 10^5^ cells per mL and 1.18 × 10^6^ ± 1.54 × 10^5^ cells per mL, respectively. Fraction D also shows a higher proportion of cells with four to six bud scars. However, a distinct bud scar number separation per outlet remains unfeasible. While the geometric adaption can reduce magnetically induced convective motion, complete elimination is unattainable. Additionally, cells continuously grow throughout their replicative life span, serving as a size separation criterion, but also exhibit size variability during their cell cycle.^4^ As illustrated in Fig. S2a,† budding and agglomerating cells have a larger diameter (14 μm) than single cells (10 μm), possibly leading to mixed age fractions. Previous studies have demonstrated the efficacy of intertial cell manipulation for microfluidic yeast cell separations.^52,55,56^ Using a pinch-shaped channel to separate yeast cells only based on morphology, a throughput of only 3.75 × 10^4^ cells per min was achieved.^52,56^ However, both fractionation methods are unsuitable for higher-scaled yeast cell fractionation or differentiation by age, only by cell groupings like singlets, duplets, and clusters.

Integrating intertial fractionation with magnetophoresis, based on age-related magnetic load, in a millifluidic pinch-shaped chip proved the most effective approach for achieving bud scar differentiation. This method has a high throughput of 1.90 × 10^6^ ± 5.71 × 10^5^ cells per min, with reproducible separation efficiencies across three independent processes: The mean and standard deviation of three independent fractionation processes is 1.00 ± 0.00 for fraction A, 0.95 ± 0.02 for fraction B, and 0.97 ± 0.00 and 0.84 ± 0.08 for fractions C and D, respectively.

### E Metabolite level changes over cell age

**Fig. 5:**
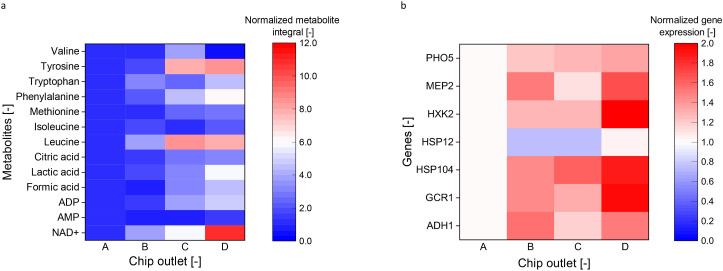
Heat map depicting the normalized integral area of selected metabolites against the cell fraction (a). Comparative analysis of gene expression across cell fractions (b). Both normalized to the cells from chip outlet A. Data included from a triplicate measurement.

Leupold *et al.* noted a decline in metabolite concentrations and growth rates with chronological aging.^16^ As shown in Fig. 5a and S8,† older cells, successfully fractionated with the pinch-shaped geometry, showed significant increases in nicotinamide adenine dinucleotide (NAD^+^) (*p*-value: 0.008), lactic acid (*p*-value: 0.015), and formic acid (*p*-value: 0.027) levels. Our study further revealed a five-fold adenosine diphosphate (ADP) accumulation between outlets D and A, reinforcing the NAD^+^ impact. The rise in lactic acid aligns with energy cofactor concepts, correlating with yeast cells' intracellular pH and adenosine triphosphatase (ATPase) activity.^57^ This suggests decreased adenosine triphosphate (ATP) cofactor levels, reduced ATPase activity, and subsequent intracellular acidification.

Cell membrane transporters internalize amino acids and are subsequently processed into alpha-keto acids by the Ehrlich pathway.^58^ These acids are either decarboxylated into aldehydes reduced to higher alcohols or oxidized to its corresponding acids before yeast cells excrete them. Among the essential amino acids, levels of leucine, methionine, phenylalanine, tryptophan, and tyrosine are lower in younger cells with an average bud scar number of 2.33 ± 0.82 in fraction A, but rise in older ones having an average bud scar number of 6.27 ± 0.45 in fraction D (*p*-value: 0.050 (leucine), 0.024 (methionine), 0.064 (phenylalanine), 0.098 (tryptophan), 0.036 (tyrosine)). Conversely, isoleucine (*p*-value: 0.298) and valine levels remain consistent throughout replicative aging (*p*-value: 0.271).

These observations can be explained by various hypotheses: (i) Rapid growth and division: Younger cells are often engaged in rapid growth and division. The lower amino acid levels might indicate increased use for protein synthesis and other cellular activities associated with growth.^25,59^ (ii) Metabolic shift: the decrease in amino acid levels within younger cells might signal a shift in their metabolism, prioritizing different pathways. These pathways might encompass energy production, nucleotide synthesis, or alcohol formation, reducing the accumulation of specific amino acids. Notably, the synthesis of esters, an energy-intensive process demanding cofactors,^60^ could be linked to this hypothesis. Consequently, the decreased amino acid levels might be due to the prevalence of ester synthesis, which often occurs during later fermentation phases. Many studies have been analyzing the metabolic pathways associated with the volatilome formation by yeast mediated fermentation, encompassing both genetic and environmental influences.^61,62^ However, an age-related perspective determined in a representative cell number remains absent. Using the proposed millifluidic chip makes it feasible to explore the interplay between age and metabolites on a scale beyond single-cell analysis.

### F Variation of cellular age on gene expression

Our study found that gene expression increases with the median cell age of yeast cells. As shown in Fig. 5b, the genes ADH1 and MEP2 showed an upregulation of around 50%. Other genes, including GCR1, HSP104, HXK2, and PHO5, were upregulated by a factor of 2 for cells having a bud scar number of 6.27 ± 0.45 compared to younger cells having a bud scar number of 2.33 ± 0.82. However, the expression of gene HSP12 did not significantly vary with the median cell age. All reference genes indicate no significant change in all four outlet samples.

These findings suggest that aging cells may experience changes in genes related to metabolism and energy regulation. For instance, genes involved in glucose metabolism and mitochondrial function may also show altered expression in other organisms. In our study, we observed an upregulation of the gene HXK2, which codes for the metabolic enzyme hexokinase,^63^ indicating a potential need for increased glycolytic activities and stress response in aging cells.^64^

The genes ADH1 (ref. 65 and 66) and GCR1 (ref. 67 snd 68) served as markers for the glycolytic pathway and ethanol formation capacity, reflecting the overall metabolic state of the cell. The expression of GCR1 increased with cell age about factor 2, with cells having an average bud scar number of 2.33 ± 0.82 showing the lowest expression. Accompanying heightened GCR1 expression, the HSP104 gene, which is involved in protein re-folding and reflects the cells' replication stress status, showed doubled expression with age. Different expression behavior of HSP104 and HSP12, of which the latter is not upregulated with cell age, is due to lack of a common activator, *i.e.* heat stress.^69^ The role of ADH1 in the formation of higher alcohols *via* the Ehrlich pathway also is consistent with the metabolite changes of the cell.

The observed changes reflect the cell's adaptive strategies to counteract the aging effects and maintain cellular functionality under stress conditions. They show upregulated genes linked to metabolism and stress response, indicating metabolic shifts and a need for heightened defenses. This upregulation, especially in genes related to glucose metabolism and mitochondrial function, underscores the impact of aging on cellular energy processes.^66,68^ Aging cells might face greater energy needs, necessitating adaptive measures for energy balance. Chen *et al.* found that glycolytic flux rises with increased growth rates,^70^ suggesting older cells may amplify glycolytic activity to meet energy requirements. Similarly, older cells might have reduced energy efficiency, compensating through heightened glycolytic activity and gene expression. Stress conditions, especially during anaerobic fermentations, can reduce growth and biomass due to energy shortages,^25^ affecting processes like aroma or protein production. Using separated young cells could offset aging effects on both genomic and metabolite levels. This approach offers potential improvements for yeast processes in the beverages and pharmaceutical industries.

## D Conclusions

We present a novel millifluidic process using magnetophoresis for age-based fractionation of *S. pastorianus* var. *carlsbergensis* culture. An optimal linker-protein to MNP ratio of *R* = 0.25 ensures homogeneous agglomerate size, enabling cell deflection in the magnetic field while reducing crosslinkage. Magnetic-induced convective motion, inherent in the process, reduces separation selectivity, necessitating a prior batch separation of daughter and mother cells. Among tested geometries, the pinch-shaped design achieved best age fractionation, increasing the average bud scar number from 2.33 ± 0.82 in fraction A to 6.27 ± 0.45 in fraction D, processing 1.90 × 10^6^ ± 5.71 × 10^5^ cells per min. This design combines magnetophoretic and inertial fractionation, with older cells having more bud scars and thus larger volumes, showing increased deflection towards the magnet. This chip, fabricated using 3D printing technology, offers an economical, high-throughput platform with high and reproducible separation selectivity, replacing single-cell microfluidic methods. Metabolomic data indicates age-related declines in specific amino acids and a rise in NAD^+^ production, possibly due to younger cells' metabolic activities. Gene expression studies highlight age-related changes, especially in metabolism and stress response genes. Aging cells seem to adjust their metabolic pathways, suggesting potential energy inefficiencies. Young cells can mitigate aging effects, benefiting the beverages and pharmaceutical industries. Our millifluidic platform introduces a non-invasive method for age-based fractionation, revolutionizing in-line age analysis in yeast processes.

## Author contributions

L. W., J. M., H. H. and M. E. – methodology, investigation, data curation. L. W., H. H. and M. E. – formal analysis. K. B. data curation. L. W. and M. E. – writing – original draft, visualization, validation, methodology. R. K. and S. S. – conceptualization, project administration, supervision. S. S., T. M., T. B. and S. B. – Resources. L. W., M. E., J. M., B. K., H. H., T. M., R. K., S. S., S. B., and T. B. – writing – review & editing.

## Conflicts of interest

There are no conflicts to declare.

## Supplementary Material

LC-024-D4LC00185K-s001
