# Supplementary material for: Millifluidic magnetophoresis-based chip for age-specific fractionation: evaluating the impact of age on metabolomics and gene expression in yeast
Source: Lab Chip. 2024 May 13;24(11):2987–98. doi: 10.1039/d4lc00185k (PMC11427765; doi:10.1039/d4lc00185k)
Supplement: LC-024-D4LC00185K-s001 [file LC-024-D4LC00185K-s001.pdf]

Electronic Supplementary Material (ESI) for Lab on a Chip.
This journal is © The Royal Society of Chemistry 2024

Electronic Supplementary information to

Millifluidic magnetophoresis-based chip for age-specific fractionation: Evaluating the impact of age on metabolomics and gene expression in yeast

Wittmann, L. [a]†, Eigenfeld, M. [b,c]†, Büchner, K.[b], Meiler, J.[a], Habisch, H.[c], Madl, T.[c,d], Kerpes, R.[b,]*, Becker, T.[c], Berensmeier, S.[a,e], Schwaminger, S. P.[a,c,d,*]

[a]Technical University of Munich, TUM School of Engineering and Design, Chair of Bioseparation Engineering, Boltzmannstr. 15, 85748 Garching, Germany

[b]Technical University of Munich, TUM School of Life Science, Chair of Brewing and Beverage Technology, Weihenstephaner Steig 20, 85354 Freising, Germany

[c]Medical University of Graz, Otto-Loewi Research Center, Division of Medicinal Chemistry, Neue Stiftingtalstr. 6, 8010 Graz, Austria

[d]BioTechMed-Graz, Mozartgasse 12/II, 8010 Graz, Austria

[e]Technical University of Munich, Munich Institute of Integrated Materials, Energy and Process Engineering, Lichtenberstr. 4a,

85748 Garching

† All authors contributed equally

*Corresponding authors:

Sebastian P. Schwaminger, E-mail: s.schwaminger@tum.de; sebastian.schwaminger@medunigraz.at

Roland Kerpes, E-mail: roland.kerpes@tum.de

A Results and Discussion

A Magnetic nanoparticle characterization.

Achieving continuous high separation selectivities necessitates the understanding of concentration and time dependence of the linker-protein@particle agglomerate formation (see Section Results A). Therefore, the nanoparticles are characterized in the following. The magnetic nanoparticles (MNP) (ethylenediaminetetraacetic acid (EDTA)- functionalized silica shell iron oxide nanoparticles) utilized for labeling the yeast cell's bud scars, as previously described [1], exhibit an iron oxide core, enabling magneto-responsive behavior. Superconducting quantum interference device (SQUID) measurements, presented in Fig. S1 a, reveal no remanence and superparamagnetic behavior, having a saturation magnetization of 13.13 emu g^{-1}. X-ray diffraction (XRD) measurements reveal that the iron oxide nanoparticles (IONs) exhibit a diameter of 13.11 ± 2.07 nm (Fig. S1 b). Further, the XRD analysis confirms the typical reflections ((220), (311), (400), (511), and (440)) for magnetite with its cubic crystal structure [2]. These iron oxide cores, evident in transmission electron microscopy (TEM) images in Fig. S1 c, are encapsulated by a silica shell that precludes the IONs from agglomerating, as previously shown [3,4]. For these particles to bind with the histidine tag of the linker-protein, an EDTA surface was introduced via an amide bond [1,5]. Although this surface is not discernible in the TEM images, it is detectable in the thermogravimetric analysis (TGA). TGA results corroborate the presence of different coatings. Fig. S1 d illustrates the weight loss across temperatures for the different coating steps. The initial weight loss step, associated with the desorption of surface-bound water, is evident at temperatures below 100°C. The iron oxide core is stable up to 700°C, with negligible weight loss. For the coatings, the weight loss between 100 and 700°C is attributed to the decomposition of functional groups, such as the carboxyl groups in the EDTA-functionalized particles. However, the thermal stability for the ION@Si and ION@Si@NH_2 particles is higher and leaves more residual [6,7]. The overall EDTA content is calculated to be 15.33 wt%. The Fourier-transform infrared spectroscopy (FT-IR) data in Fig. S1 e confirms the different particle functionalization steps. The Fe-O-Si vibration, at

592 cm^{-1}, verifies the binding of the silica to the ION core, besides the Si-O-Si stretching vibration at 1080 cm^{-1} and the Si-O vibration at 794 cm^{-1}. The successful functionalization with amine groups is confirmed by the 1488 and 1638 cm^{-1} bands, which are associated with the N-H bending vibrations. This is indicative of the effective incorporation of (3-aminopropyl)triethoxysilane (APTES) [8]. EDTA was coated on the particles in an additional synthesis step, proved by the asymmetrical and symmetrical COO^- stretching vibrations, located at 1570 – 1610 cm^{-1} and 1350 – 1450 cm^{-1}, respectively. Furthermore, the disappearance of the N-H bending vibration at 1488 cm^{-1} signifies the successful establishment of an amide bond between APTES and EDTA [5]. The primary particle size from TEM analysis is 120.62 ± 16.86 nm (compare Fig. S1 c and f). After synthesis, dynamic light scattering (DLS) measurements yielded a hydrodynamic diameter of 2297.67 ± 850.78 nm (ζ = -8.22 ± 0.39 mV, pH = 9.1) for the ION@Si@NH_2 precursor and 179.01 ± 11.99 nm (ζ = 21.10 ± 0.62 mV, pH = 4.89) for the EDTA-functionalized MNPs with a polydispersity index of 0.17 ± 0.02 (Fig. S1 g). These results confirm the colloidal stability and monomodal distribution of the built ION@Si@EDTA particles (the term 'Si' refers to the silica shell). Moreover, the hydrodynamic diameter can be preserved for up to 28 days, as confirmed by an agglomeration study in Fig. S1 h. After this period, the hydrodynamic diameter measures 176.60 ± 32.55 nm, with a zeta potential of -29.26 ± 1.53 mV. The EDTA-functionalized surface enables the binding of the linker-protein via the His-tag. Therefore, this surface is saturated with nickel (Ni) ions, achieving maximum binding capacities of 4 $mg_{Ni}\ g_{Particle}^{-1}$ (Fig. S1 i), corresponding to 0.40 nickel ions per $nm_{Particle}^2$, comparable to Fraga García et al. [9]. The binding kinetics occurs within minutes (Fig. S1 j), a finding that aligns with previous studies [10], and the built EDTA@Ni chelate complex remains sTab. for several days, as no leaching was observed over five days, presented in Fig. S1 k. The differences in surface charges for the precursor particles, the EDTA-functionalized ones, and those saturated with nickel ions were investigated across a range of pH values in water. The study reveals that the isoelectric point (IEP) of the ION@Si@NH_2 is at pH 8.8 and decreases to pH 6.8 for the ION@Si@EDTA (Fig. S1 l). The presence of loaded nickel ions slightly reduces the IEP to 7.6. The particles are colloidally sTab. (ζ > I 20 mV I) or unsTab. (-20 mV < ζ > 20 mV), dependent on the surface charge. This pH-dependent agglomeration directly influence the sedimentation velocity under a magnetic field gradient, as Fig. S1 m shows. After the synthesis, the ION@Si@NH_2 particles exhibit a pH of 9.1, close to the IEP, leading to rapid sedimentation in a magnetic field. Contrarily, the ION@Si@EDTA exhibit a pH of 4.89 after the synthesis, further away from their IEP. Thus, they tend to sediment slower in the magnetic field due to the formation of smaller agglomerates. These findings emphasize the importance of carefully controlling the agglomeration behavior for the subsequent magnetophoretic fractionation process (Fig. S1 m).

Consequently, adhering to the established particle dispersion protocol for each iteration of fractionation experimentation became important. The MNPs, stored at a temperature of 4°C under a nitrogen atmosphere, exhibit an average hydrodynamic diameter of 306.73 ± 7.96 nm, accompanied by a polydispersity index of 0.32 ± 0.1. The MNP solution underwent ultrasonication to disperse larger agglomerates, which diminishes the polydispersity index to 0.13 ± 0.01 (d_{hyd} = 226.57 ± 0.85 nm). Consequently, this ensures the use of relatively homogeneous agglomerates for the nickel loading procedure. Post-loading, the hydrodynamic diameter increases to approximately 1000 nm, as the nickel-loaded MNPs arre resuspended in the buffer utilized for incubation with the linker-protein labeled yeast cells (Fig. S1 n).

The process of nickel loading is notably time-intensive, requiring approximately two hours. Improving the efficiency of the overall labeling process, we assessed whether nickel-loaded particles could be stored for several days and subsequently reused. This involved monitoring the hydrodynamic diameter over a three-day storage period, as presented in Fig. S1 o. The findings suggest that a consistent hydrodynamic diameter cannot be maintained, as the distribution indicates a reduction in the hydrodynamic diameter throughout the storage. This reduction implies that, even during storage, the agglomerates persist in achieving equilibrium within the buffer. Consequently, the MNPs were freshly loaded with nickel ions before each fractionation iteration to ensure reproducible agglomerate sizes.

B Homogenization of magnetically labeled yeast cells during the fractionation process

Maintaining a consistent sample concentration flowing into the chip was essential in optimizing the magneto-responsive fractionation process since the magnetic dipole moment modulates the magnetophoretic force acting upon the labeled yeast cells. This moment is a function of both concentration and agglomerate size, as described in Equation 1.

The brewing industry uses top-fermenting *S. cerevisiae* and bottom-fermenting *S. pastorianus* var. *carlsbergensis* for ale and lager production. Both encompass strains that flocculate or remain single cells, influencing the aroma formation and reuse after fermentation [11,12]. The sedimentation rate is primarily influenced primarily by the strain's flocculation characteristics within minutes [13,14]. Although the strain employed in this study was non-flocculating, consisting mainly of single cells or budding cells (Fig. 2 f and S2 a), they tend to sediment due to buoyancy depending on the cell (agglomerate) size (Fig. S2 c). Fig. S3 a illustrates the sedimentation behavior of yeast cells within the sample syringe before their introduction into the millifluidic chip. Notably, the yeast cells exhibited significant sedimentation without any dispersion method (when stationary). Within 20 min, they settled to a proportion 0.27 ± 0.4 of their initial count, leading to an inhomogeneous yeast concentration during fractionation. Consequently, a

dispersion method ensures homogeneity throughout the fractionation process. Previous studies used conventional magnetic stirring for mixing inside the syringe [15], but these were incompatible due to their interference with the MNPs. Therefore, alternative dispersion strategies were explored. This sphere facilitates direct mechanical mixing inside the syringe when positioned on the rocker. As presented in Fig. S3 a, the shaker does not significantly mitigate sedimentation, especially at higher mixing rates, which induce turbulences within the chip. The rocker results in better dispersion but with variability, maintaining between a proportion of 0.77 ± 0.12 and 0.93 ± 0.15 of the initial yeast cell number. Introducing a sphere within the syringe emerges as the most efficient approach. With the sphere's motion enabled by the teetering rocker, yeast cells are uniformly dispersed, preserving nearly 100 % of the initial yeast cell number. Importantly, this method does not comprise yeast cell viability: 75 ± 14 % of cells are viable pre-insertion and 79 ± 13 % post-process without affecting the magnetic labeling of the yeast cells (Fig. S2 b).

C Magnetic aggregation and magnetically induced convection

The cooperative motion is quantified by the dimensionless aggregation parameter N^*, influenced by the volume fraction Φ_0 in the solution, and the magnetic coupling parameter Γ are expressed as,

$$N^* = \sqrt{\Phi_0\, e^{(\Gamma - 1)}} \tag{S1}$$

$$\Gamma = \frac{\mu_0 m^2}{2\pi d^3 k_B T} \tag{S2}$$

Where μ_0= 4 π x 10^{-7} N A^{-2} is the magnetic permeability of free space, $\vec{m}$ the induced magnetic dipole moment, k_B = 1.38 x 10^{-23} J K^{-1} is Boltzmann's constant and T is the absolute temperature. In line with the observations made by Leong et al., field-induced self-assembly of particles is not anticipated to occur when the dimensionless aggregation parameter is N^* < 1.

The second dimensionless number, characterizing the motion of MNPs when exposed to a magnetic field, is the magnetic Grashof number Gr_m, accounting for the induced convective motion, given by

$$Gr_m = \frac{\rho \nabla B \left(\frac{\partial M}{\partial c}\right)_H (c_s - c_\infty) L_c^3}{\eta^2} \tag{S3}$$

Where ρ is the density, $\vec{\nabla} B$ is the magnetic field gradient within the magnetic field strength B, M is the volumetric magnetization in solution. c_s is the particle concentration at the collection plane, c_∞ is the one in the sample solution, and L_c is the characteristic length of the system subjected to magnetophoresis, which is the chip width in our case.

The following values are used for calculating the magnetic Grashof number and the aggregation parameter:

Magnetization at 0.38 T [A m^{-1}]	28450.88
Particle diameter d [m]	9.68×10^{8}
Particle volume [m^{-3}]	4.75×10^{22}
Volume all particles in system [m^3]	3.89×10^{6}
Density particles, 22°C [g L^{-1}]	2800.80
Density water, 22°C [g L^{-1}]	997.77
Density system ρ [g L^{-1}]	998.03
Particle volume fraction φ	0.00015
Coupling parameter Γ	1.36×10^{28}
Magnetic field gradient at collection surface ∇B [T m^{-1}]	4.82
Volumetric magnetization of solution M [(A m^{-1}) (g L^{-1})$^{-1}$]	10.16
Particle concentration at collection plane c_s [g L^{-1}]	0
Particle concentration of bulk solution c_∞ [g L^{-1}]	0.4
Characteristic length of system L_c [m]	0.00313
Viscosity η [kg ms^{-1}]	0.00010
Chip volume [m^3]	0.02721

B Figures and Tables

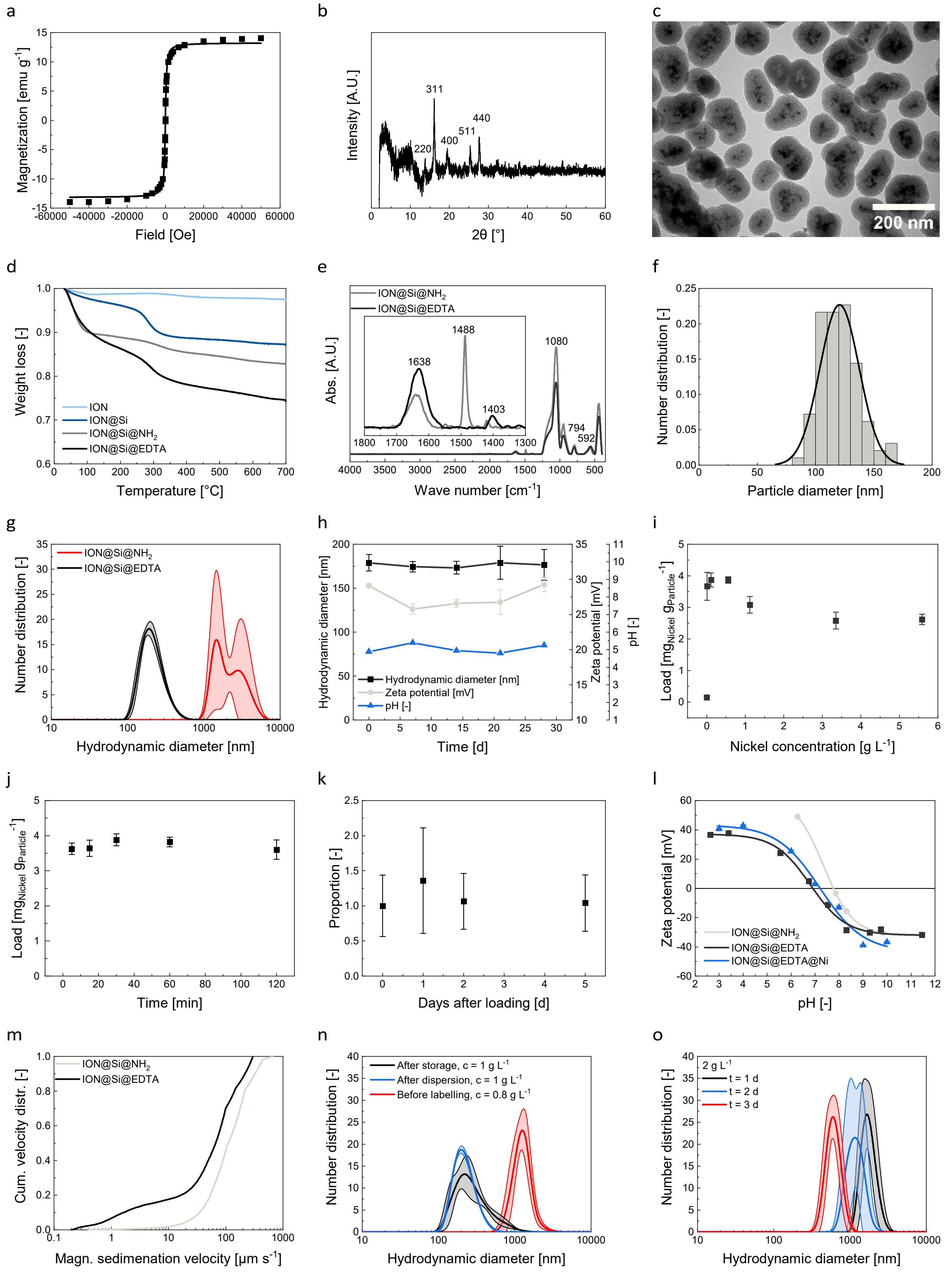

Fig. S1 Superconducting quantum interference device (SQUID) measurement (a), X-ray diffractogram (b), and transmission electron microscopy (TEM) images (c) of the final ethylenediaminetetraacetic acid (EDTA)-functionalized silica-coated IONs. Thermogravimetric analysis (d) of the different coating steps (the term 'Si' refers to the silica shell). Fourier-transform infrared (FT-IR) spectroscopy analysis of the precursor 3-aminopropyltriethoxysilan (APTES)-coated in grey and EDTA-functionalized IONs in black (e). Number distribution over particle diameter derived from ImageJ analysis of the TEM images for n = 100 MNPs (f). Dynamic light scattering measurement (DLS) of the precursor APTES-coated (pH = 9.1) and final EDTA-functionalized IONs (pH = 4.89) after synthesis in water for c = 1 g L^{-1} (g). Hydrodynamic diameter, zeta potential, and pH for the MNPs (the term MNP refers to the final EDTA-functionalized silica shell iron oxide nanoparticles) up to 28 days, stored under nitrogen at 4°C (h). Nickel binding isotherm to MNPs for various nickel concentrations ($c_{Particles}$ = 1 g L^{-1}) (i). Binding kinetics for nickel binding (c_{Nickel} = 0.59 g L^{-1}) to the MNPs ($c_{Particles}$ = 1 g L^{-1}) (j). The nickel leaching study shows the proportion of the initial nickel load after several days of storage at 4°C

(k). Zeta potential for the precursor APTES-coated and final EDTA-functionalized IONs and those saturated with nickel ions for different pH values ($c_{Particles}$ = 1 g L^{-1}) (l). Cumulative velocity distribution over the magnetophoretic sedimentation velocity for the precursor APTES-coated (pH = 9.1) and final EDTA-functionalized (pH = 4.89) IONs in water after synthesis ($c_{Particles}$ = 1 g L^{-1}) (m). DLS measurement of the MNP solution in water after storage (black), after dispersion (blue) before the nickel loading, and before labeling, but after nickel loading (red) in the necessary concentrations of the protocol (n). Number distribution of the DLS measurement for a storage study of the nickel-loaded MNPs in buffer over three days (o). Error bars or shaded areas represent the standard deviation of a triplicate measurement.

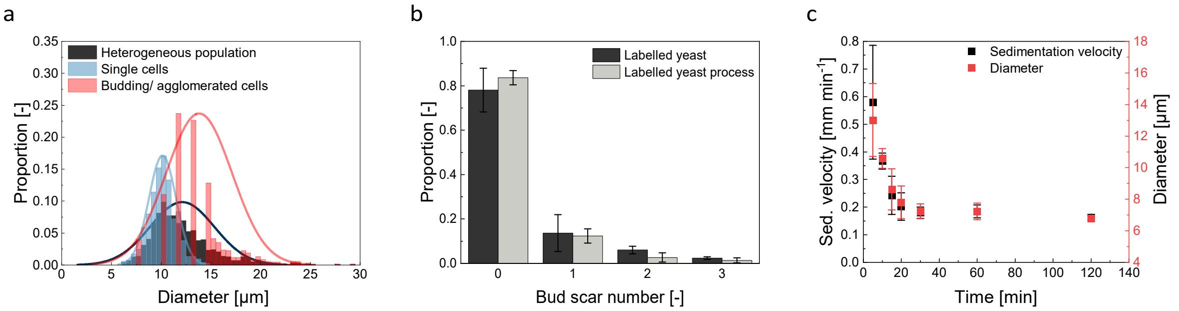

Fig. S2 Yeast cell diameter of a heterogeneous population derived from ImageJ analysis of n = 1168 cells containing 45 % single cells and 55 % buddying/ agglomerated cells (a). Age distribution of a magnetically labeled heterogeneous yeast culture derived from cytometric analysis (n_{min} = 20000 counts, n = 3, error bars representing the standard deviation of the triplicate measurement) before the process (black) and in during the process with the dispersion via the rocker and the sphere (b). Sedimentation velocity and corresponding yeast cell (agglomerate) diameter over time at 410 nm (c). Error bars represent the standard deviation of a triplicate measurement.

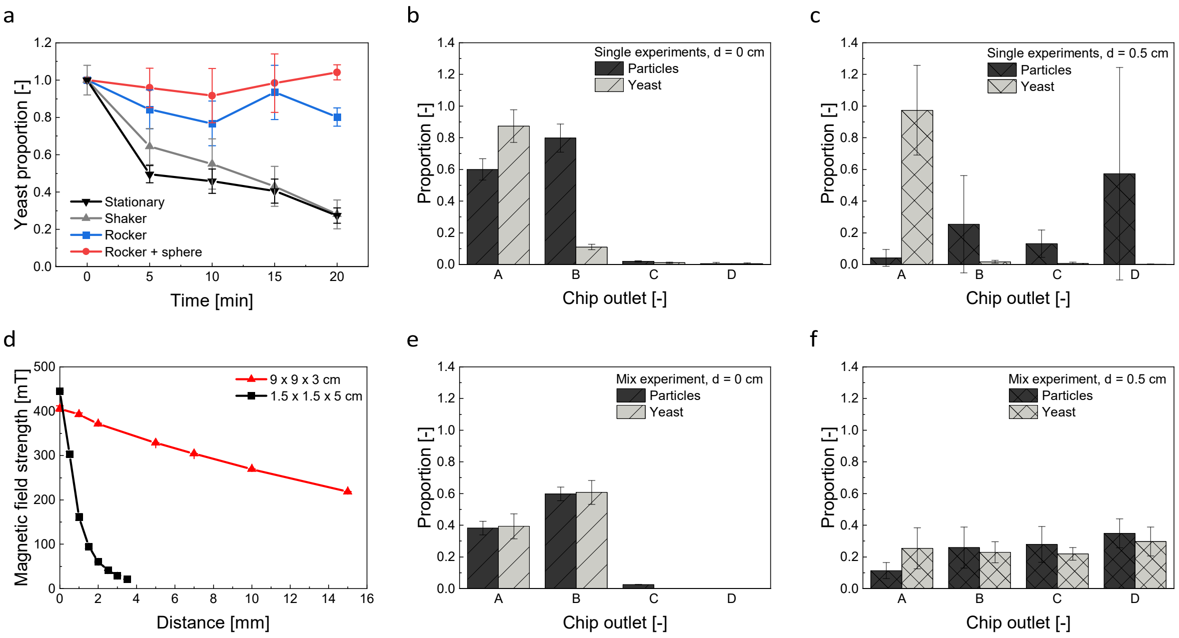

Fig. S3 Yeast proportion of the initial cell number of $1.58x10^{7}$ cells mL^{-1} in sample syringes over time for different dispersion methods using no dispersion method (stationary), a shaker, a rocker, and an inserted sphere in the sample syringe positioned on the rocker to avoid sedimentation (a). Single fractionation experiments for solely yeast and particles (b) and (c) and for yeast-particle mixture without linker-protein (e) and (f) in the rectangular geometry. Chip outlet A was the furthest away from the magnet; chip outlet D was nearest to the magnet. $\dot{V}_{Sample}$ = 220 µL min^{-1}, $\dot{V}_{Buffer}$ = 1840 µL min^{-1}, the magnet distance was 0 cm (a) and (d). $\dot{V}_{Sample}$ = 220 µL min^{-1}, $\dot{V}_{Buffer}$ = 920 µL min^{-1}, the magnet distance was 0.5 cm, $c_{Particle}$ = 0.4 g L^{-1}, cell number = $1.59x10^{7}$ cells mL^{-1} (b) and (e). Measured magnetic field strength using an AC/DC magnetometer (PCE Instruments) over the distance from the magnet surface at the middle for the used magnets (9 x 9 x 3 cm for the rectangular and 1.5 x 1.5 x 5 cm for the trapezoidal geometry) (d). All data plots include the standard deviation from a triplicate measurement.

a

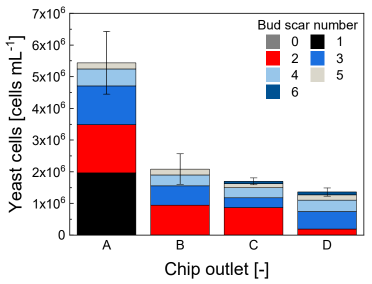

b

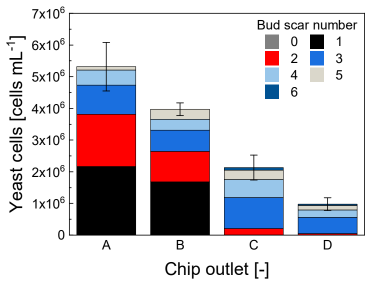

Fig. S4: Reproduction of the yeast cell fractionation with the pinch-shaped geometry. Yeast cell concentrations for the different fractions and corresponding bud scar numbers are shown. $c_{Particle}$ = 0.4 g L^{-1}, cell number = 1.59x10^7 cells mL^{-1}. Error bars represent the standard deviation of the triplicate measurement. Outlet A was the furthest away from the magnet; outlet D was the nearest to the magnet. Daughter and mother cells refer to the prior batch separation.

a

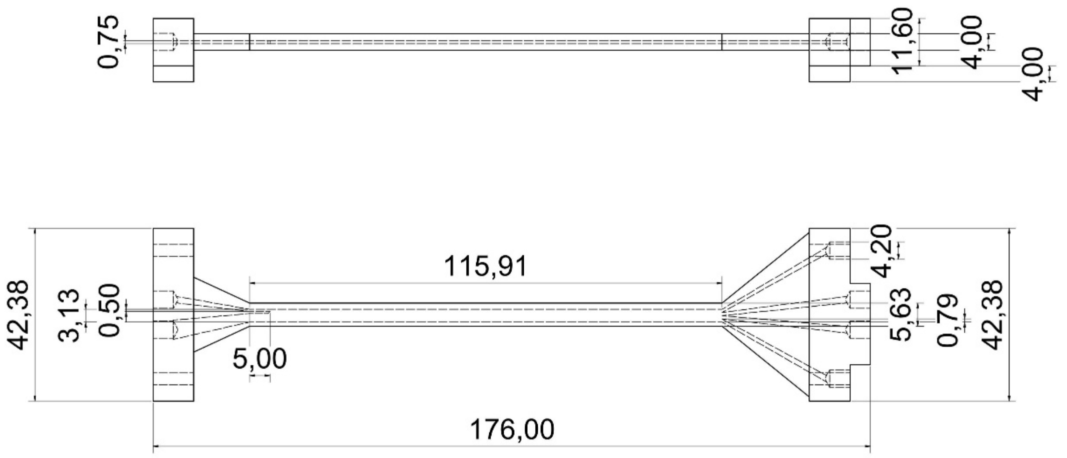

b

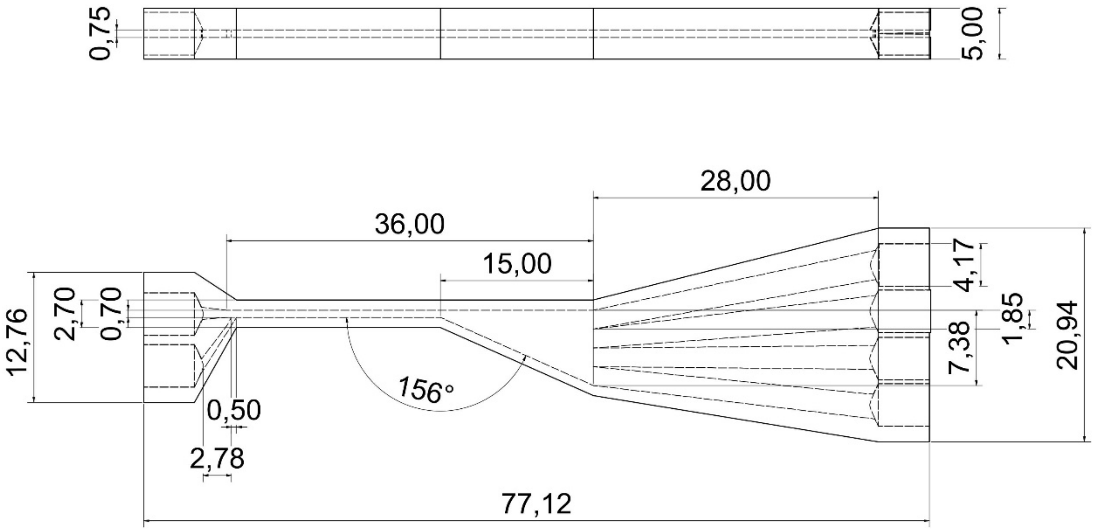

c

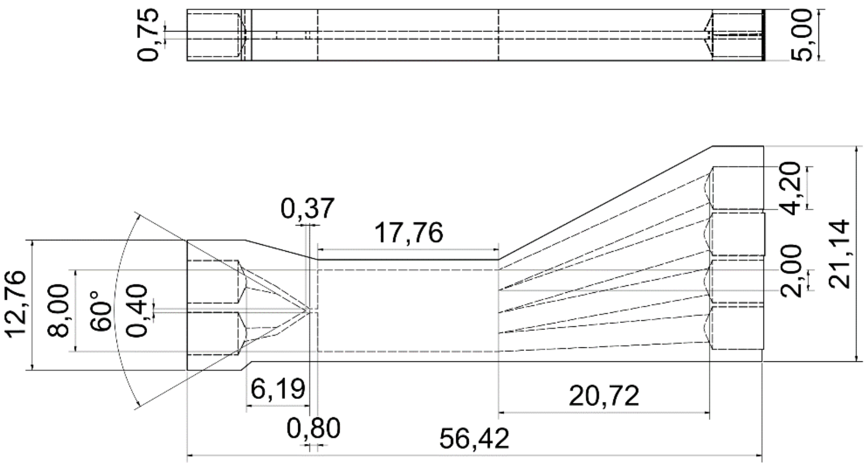

Fig. S5 Technical drawings in [mm] of the rectangular (a), trapezoidal (b), and pinch-shaped (c) chip.

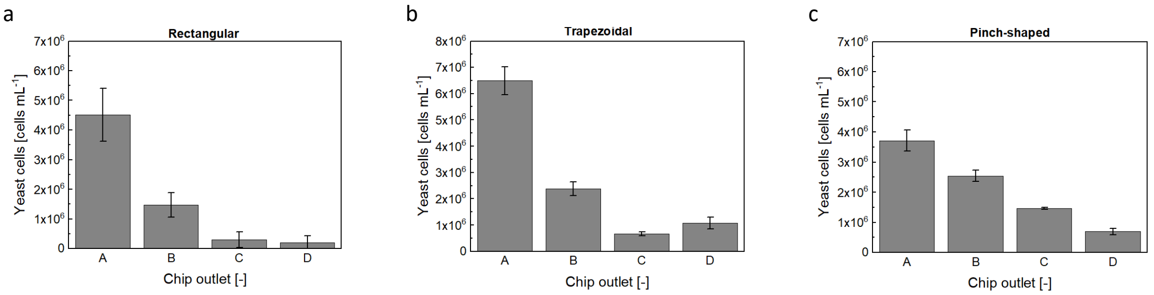

Fig. S6: Yeast cell concentration for different fractionations with different flow channels (rectangular (a), trapezoidal (b), pinch-shaped (c)). The yeast cells were not labeled with linker-protein or MNPs. Error bars represent the standard deviation of the triplicate measurement. Outlet A was the furthest away from the magnet; outlet D was the nearest to the magnet.

Tab. S1: Separation selectivities for the different chip geometries derived from the cumulative bud scar number distribution related to the cell number in each chip outlet. The rectangular and trapezoidal geometry fractionation was performed once, and the pinch geometry process was conducted three times.

Chip outlet	Bud scar number n	Rectangular	Trapezoidal	Pinch
A	≥ 1	0.48	0.48	1.00 ± 0.00
B	≥ 2	0.94	0.95	0.95 ± 0.02
C	≥ 3	0.96	0.40	0.97 ± 0.00
D	≥ 4	0.66	0.34	0.84 0.08

C Methods

A Comparison between microscopic determined bud scar distribution and flow cytometric analysis.

To ensure the reliability of flow cytometric results on bud scar distribution in heterogeneous yeast populations and associated outlet distributions, we proposed two distinct measurement methodologies, described earlier in detail [1,16,17]:

1. Microscopy-based approach:

This involved manual counting of bud scars via confocal and light microscopy and subsequent predictions using the Bayes theorem. The method followed the protocol by Eigenfeld et al. [17], which postulates that only 40% of the yeast cell surface was observable through microscopy. Consequently, predictions had to be made for the remaining 60% of the cell surface where bud scars were unobservable.

1. Flow cytometric approach [16]:
    a. Outlier elimination using the R package 'mvoutlier'.

b. Autofluorescence predictions via the Random Forest algorithm.
c. Single-cell autofluorescence subtraction from each yeast cell.
d. Analysis of the resultant fluorescence signals via Gaussian histogram curve fitting (Fig. S6). Notably, each Gaussian curve corresponds to an age cluster, symbolizing cells with an identical count of bud scars.

This comparative study aids in validating the flow cytometric determinations against a microscopy-based reference. The results showed no noTab. differences between the two measurement methods in terms of heterogeneous populations (p-value = 0.9375), mother cells (p-value = 0.8125), and daughter cells (p-value = 0.5781) when a Wilcoxon paired test was employed. Furthermore, through the automated Gaussian mixture analysis provided by the R package 'GaussianMixture', we found p-values of 1 for both heterogeneous populations and mother cells and a p-value of 0.4609 for daughter cells.

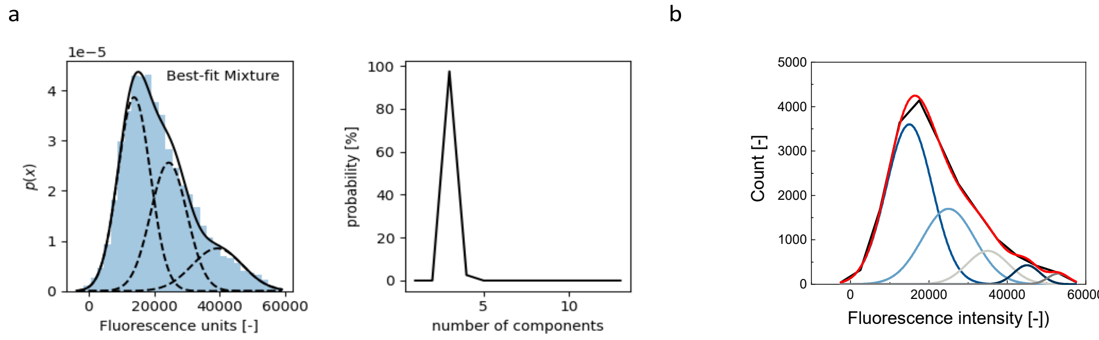

Fig. S7 Gaussian mixture analysis using R package 'GaussianMixture' (a) and using OriginLabs 2021 (R1 = 0.9995) (b) for a heterogeneous population.

B Primer sequences for quantitative polymerase chain reaction experiments

Tab. S2: Primer sequences of ones used to amplify the housekeeping genes.

TAF10	forward primer	TAACAACAGTCAGGCGAGAG
	reverse primer	CACCGTCAGAACAACTTTGC
KRE11	forward primer	ATTCGCCCTTGACACTGG
	reverse primer	CTCTCGGAGGTACAACTG
UBC6	forward primer	ATGCGGCAAATACAGGTGATG
	reverse primer	TTGTTCAGCGCGTATTCTGTC

Tab. S3: Primer sequences of the tested genes.

Gene		Primer sequence
MEP2	forward primer	ACGAGGAATCCACTGCTTAC
	reverse primer	CGTCTGTGTTACCCACAATC
HSP104	forward primer	TAACTCAAGAGGCCAAGGAC
	reverse primer	TCCTTAGTGCCAGTTTGTTC
HXK2	forward primer	GGCTGCCAATGCTTTGAAGG
	reverse primer	ACCGGAACCATCTTCAGCAG
HSP12	forward primer	CTCTGCCGAAAAAGGCAAGG
	reverse primer	GACGGCATCGTTCAACTTGG
PHO5	forward primer	TTCAACATCACCTTGCAGAC
	reverse primer	ATTGGCATCGTAGTCCCAAG
ADH1	forward primer	GTGCTCACGGTGTCATCAAC
	reverse primer	GCATACCGACCAAAACGGTG
GCR1	forward primer	CCAAACAACGACTCCACTAC
	reverse primer	ATCATTGGGCTCCGACTTAC

C Minimum information for publication of quantitative real-time polymerase chain reaction experiments guidelines

Tab. S4: Minimum information for publication of quantitative real-time polymerase chain reaction experiments (MIQE) checklist for authors, reviewers, and editors. All essential information (E) must be submitted with the manuscript. Desirable information (D) should be submitted if available. If using primers obtained from RTPrimerDB, information on quantitative polymerase chain reaction (qPCR) target, oligonucleotides, protocols, and validation was available from that source.

ITEM TO CHECK	IMPORTANCE	CHECKLIST
**EXPERIMENTAL DESIGN**		
Definition of experimental and control groups	E	control group: Sample A, youngest fraction
Number within each group	E	3 Biological replicates
Assay carried out by core lab or investigator's lab?	D	
Acknowledgement of authors' contributions	D	
**SAMPLE**		
Description	E	Yeast cells, separated for cell age
Volume/mass of sample processed	D	
Microdissection or macrodissection	E	not applicable
Processing procedure	E	After separation, cells were submerged in 1/3 vol. 5% phenol in abs. ethanol and frozen
If frozen - how and how quickly?	E	
If fixed - with what, how quickly?	E	5 % Phenol in absolute Ethanol, 1:3 with liquid sample, directly after cell fractionation
Sample storage conditions and duration (especially for FFPE samples)	E	Samples were stored at -80°C in 2/3 separation buffer and 1/3 vol. 5% phenol in abs. ethanol
**NUCLEIC ACID EXTRACTION**		
Procedure and/or instrumentation	E	Enzymatic cell lysis with subsequent spin column extraction
Name of kit and details of any modifications	E	Roboklon Universal RNA Kit, Roboklon GmbH, Berlin, Germany
Source of additional reagents used	D	
Details of DNase or RNAse treatment	E	None, as per protocol
Contamination assessment (DNA or RNA)	E	Denaturing RNA gel electrophoresis for degradation, no-reverse-transcription control in the qPCR
Nucleic acid quantification	E	Sheet_3
Instrument and method	E	Nanodrop 2000c spectrophotometer
Purity (A260/A280)	D	
Yield	D	
RNA integrity method/instrument	E	MOPS-Gel electrophoresis
RIN/RQI or Cq of 3' and 5' transcripts	E	not applicable
Electrophoresis traces	D	
Inhibition testing (Cq dilutions, spike or other)	E	not applicable due to small sample volume

REVERSE TRANSCRIPTION		
Complete reaction conditions	E	Thermo Scientific™ Maxima H Minus Reverse Transcriptase, according to manufacturer protocol, with 200 U of enzyme per reaction
Amount of RNA and reaction volume	E	1849.5 ng/µL, Samples 6: 810 ng/µL, Samples 7: 483.3 ng/µL; rection volume 20 µL
Priming oligonucleotide (if using GSP) and concentration	E	NEB Oligo dT VN
Reverse transcriptase and concentration	E	Thermo Scientific™ Maxima H Minus Reverse Transcriptase, 200 U of enzyme per reaction
Temperature and time	E	30 min at 50°C
Manufacturer of reagents and catalog numbers	D	
Cqs with and without RT	D*	
Storage conditions of cDNA	D	
qPCR TARGET INFORMATION		
If multiplex, efficiency and LOD of each assay.	E	not applicable
Sequence accession number	E	Sheet_2
Location of amplicon	D	
Amplicon length	E	Sheet_2
In silico specificity screen (BLAST, etc.)	E	Primer BLAST, https://www.ncbi.nlm.nih.gov/tools/primer-blast/
Pseudogenes, retropseudogenes, or other homologs?	D	
Sequence alignment	D	
Secondary structure analysis of amplicon	D	
Location of each primer by exon or intron (if applicable)	E	only exons targeted
What splice variants were targeted?	E	not applicable
qPCR OLIGONUCLEOTIDES		
Primer sequences	E	Sheet_2
RTPrimerDB Identification Number	D	
Probe sequences	D**	
Location and identity of any modifications	E	not applicable
Manufacturer of oligonucleotides	D	
Purification method	D	
qPCR PROTOCOL		

Complete reaction conditions	E	Biozym Blue S’Green qPCR Mix Separate ROX
Reaction volume and amount of cDNA/DNA	E	10 µL
Primer (probe), Mg++ and dNTP concentrations	E	Primer conc. 400 nM each
Polymerase identity and concentration	E	Manufacturer Master Mix
Buffer/kit identity and manufacturer	E	Biozym Blue S’Green qPCR Mix Separate ROX, Biozym Scientific GmbH
Exact chemical constitution of the buffer	D	
Additives (SYBR Green I, DMSO, etc.)	E	SYBR Green
Manufacturer of plates/tubes and catalog number	D	
Complete thermocycling parameters	E	Initial denaturation: 2 min, 95°C; 45x 5 sec, 95°C, 30 sec, 60°C
Reaction setup (manual/robotic)	D	
Manufacturer of qPCR instrument	E	Roche LightCycler 480 II
qPCR VALIDATION		
Evidence of optimization (from gradients)	D	
Specificity (gel, sequence, melt, or digest)	E	Melting curve, single peaks for all reactions
For SYBR Green I, Cq of the NTC	E	37.11
Standard curves with slope and y-intercept	E	
PCR efficiency calculated from slope	E	
Confidence interval for PCR efficiency or standard error	D	
r2 of standard curve	E	
Linear dynamic range	E	
Cq variation at lower limit	E	
Confidence intervals throughout range	D	
Evidence for limit of detection	E	
If multiplex, efficiency and LOD of each assay.	E	not applicable
DATA ANALYSIS		
qPCR analysis program (source, version)	E	LightCycler 480 Software release 1.5.0; qbase+, V3.4
Cq method determination	E	Proprietary Roche LighCycler
Outlier identification and disposition	E	according to qbase+
Results of NTCs	E	

Justification of number and choice of reference genes	E	according to geNorm V and geNorm M
Description of normalization method	E	Modified Pfaffl Method, according to Hellemans et al., implemented in qbase+
Number and concordance of biological replicates	D	
Number and stage (RT or qPCR) of technical replicates	E	technical duplicates in the qPCR
Repeatability (intra-assay variation)	E	not applicable
Reproducibility (inter-assay variation, %CV)	D	
Power analysis	D	
Statistical methods for result significance	E	One-way ANOVA
Software (source, version)	E	Biogazelle qbase+, V3.4
Cq or raw data submission using RDML	D	

*: Assessing the absence of DNA using a no RT assay was essential when first extracting RNA. Once the sample had been validated as RDNA-free, the inclusion of a no-RT control was desirable but no longer essential.

**: Disclosure of the probe sequence was highly desirable and strongly encouraged. However, since not all commercial pre-designed assay vendors provide this information, it cannot be an essential requirement. Use of such assays was advised against.

D Data analysis of the metabolome and gene expression

In Fig. 5, the metabolome and gene expression data are normalized to outlet A of the pinch shaped fractionation, and not to the daughter cells. This distinction is essential, as the daughter cells were separated prior to the millifluidic fractionation to avoid the magnetically induced co-migration. Consequently, direct comparisons between the separated daughter cells and the older fractionated cells are not feasible for two reasons. First, the daughter cells were not fractionated in the chip and, therefore, did not undergo 'sorting stress.' Second, they were fixed at varying time points due to the multi-step experimental process.

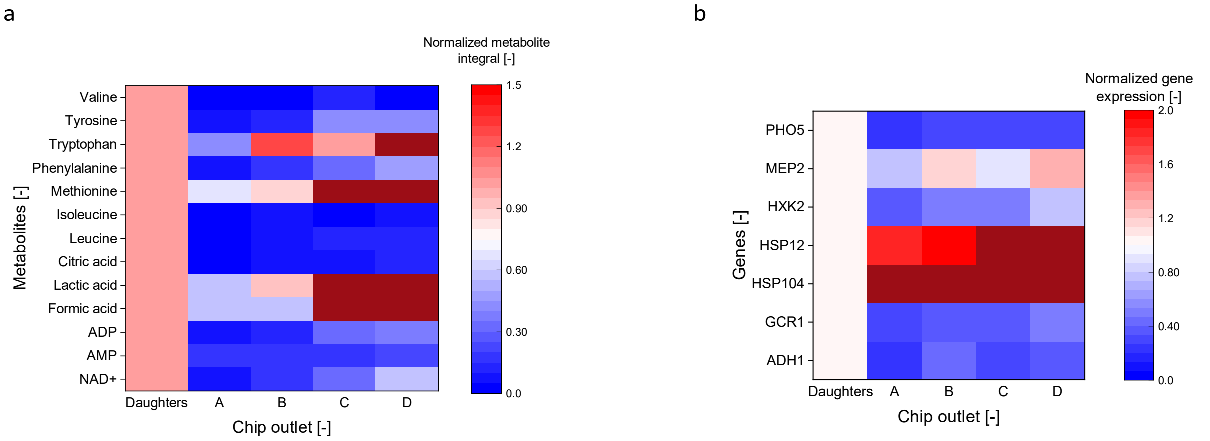

Fig. S8 Heat map depicting the normalized integral area of selected metabolites (a) and gene expression (b) against the cell fraction. Normalized to the daughter cells. Data included from a triplicate measurement.

References

1 M. Eigenfeld, L. Wittmann, R. Kerpes, S. P. Schwaminger and T. Becker, *Biotechnol. J.*, , DOI:10.1002/biot.202200610.
2 C. Turrina, S. Berensmeier and S. P. Schwaminger, *Pharmaceuticals*, , DOI:10.3390/ ph14050405.
3 S. E. Favela-Camacho, E. J. Samaniego-Benítez, A. Godínez-García, L. M. Avilés-Arellano and J. F. Pérez-Robles, *Colloids Surfaces A Physicochem. Eng. Asp.*, 2019, **574**, 29–35.
4 L. Wittmann, C. Turrina and S. P. Schwaminger, *Magnetochemistry*, 2021, **7**, 80.
5 D. Salman, T. Juzsakova, M. A. Al-Mayyahi, R. Akos, S. Mohsen, R. I. Ibrahim, H. D. Mohammed, T. A. Abdullah, E. Domokos and T. Korim, *J. Phys. Conf. Ser.*, 2021, **1773**, 0–12.
6 Y. Liu, R. Fu, Y. Sun, X. Zhou, S. A. Baig and X. Xu, *Appl. Surf. Sci.*, 2016, **369**, 267–276.
7 I. Dindarloo Inaloo, S. Majnooni, H. Eslahi and M. Esmaeilpour, *Appl. Organomet. Chem.*, 2020, **34**, 1–17.
8 T. S. Hamidon and M. H. Hussin, *Prog. Org. Coatings*, 2020, **140**, 105478.
9 P. Fraga García, M. Freiherr Von Roman, S. Reinlein, M. Wolf and S. Berensmeier, *ACS Appl. Mater. Interfaces*, 2014, **6**, 13607–13616.
10 J. Chen, Y. Hao and M. Chen, *Environ. Sci. Pollut. Res.*, 2014, **21**, 1671–1679.
11 G. G. Stewart, *Fermentation*, , DOI:10.3390/fermentation4020028.
12 K. J. Verstrepen, G. Derdelinckx, H. Verachtert and F. R. Delvaux, *Appl. Microbiol. Biotechnol.*, 2003, **61**, 197–205.
13 E. V. Soares and M. Mota, *J. Inst. Brew.*, 1997, **103**, 93–98.
14 M. Stratford, *Adv. Microb. Physiol.*
15 R. Sánchez, B. Horstkotte, K. Fikarová, H. Sklenářová, S. Maestre, M. Miró and J. L. Todolí, *Anal. Chem.*, 2017, **89**, 3787–3794.
16 M. Eigenfeld, R. Kerpes, I. Whitehead and T. Becker, *Biotechnol. J.*, , DOI:10.1002/biot.202200091.
17 M. Eigenfeld, R. Kerpes and T. Becker, *RSC Adv.*, 2021, **11**, 31923–31932.
